# Effect of Lysyl Oxidase Inhibition on Angiotensin II-Induced Arterial Hypertension, Remodeling, and Stiffness

**DOI:** 10.1371/journal.pone.0124013

**Published:** 2015-04-13

**Authors:** Lance S. Eberson, Pablo A. Sanchez, Beenish A. Majeed, Supannikar Tawinwung, Timothy W. Secomb, Douglas F. Larson

**Affiliations:** 1 Department of Physiology, The University of Arizona, Tucson, Arizona, United States of America; 2 Department of Pharmacology, The University of Arizona, Tucson, Arizona, United States of America; 3 Sarver Heart Center, College of Medicine, The University of Arizona, Tucson, Arizona, United States of America; Boston University Goldman School of Dental Medicine, UNITED STATES

## Abstract

It is well accepted that angiotensin II (Ang II) induces altered vascular stiffness through responses including both structural and material remodeling. Concurrent with remodeling is the induction of the enzyme lysyl oxidase (LOX) through which ECM proteins are cross-linked. The study objective was to determine the effect of LOX mediated cross-linking on vascular mechanical properties. Three-month old mice were chronically treated with Ang II with or without the LOX blocker, β -aminopropionitrile (BAPN), for 14 days. Pulse wave velocity (PWV) from Doppler measurements of the aortic flow wave was used to quantify in vivo vascular stiffness in terms of an effective Young’s modulus. The increase in effective Young’s modulus with Ang II administration was abolished with the addition of BAPN, suggesting that the material properties are a major controlling element in vascular stiffness. BAPN inhibited the Ang II induced collagen cross-link formation by 2-fold and PWV by 44% (P<0.05). Consistent with this observation, morphometric analysis showed that BAPN did not affect the Ang II mediated increase in medial thickness but significantly reduced the adventitial thickness. Since the hypertensive state contributes to the measured in vivo PWV stiffness, we removed the Ang II infusion pumps on Day 14 and achieved normal arterial blood pressures. With pump removal we observed a decrease of the PWV in the Ang II group to 25% above that of the control values (P=0.002), with a complete return to control values in the Ang II plus BAPN group. In conclusion, we have shown that the increase in vascular stiffness with 14 day Ang II administration results from a combination of hypertension-induced wall strain, adventitial wall thickening and Ang II mediated LOX ECM cross-linking, which is a major material source of vascular stiffening, and that the increased PWV was significantly inhibited with co-administration of BAPN.

## Introduction

Aortic stiffness is an independent risk factor for an array of cardiovascular, renal, and cerebral diseases. Increased aortic stiffness is paralleled by compositional changes of the vascular extracellular matrix (ECM), vascular smooth muscle cell (VSMC) cytoskeletal proteins [1] and physiological changes in VSMC tone. Experimental and clinical data strongly suggest that altered blood flow patterns secondary to aortic stiffness are initiating events of the above mentioned disease conditions. It is therefore logical to examine the relationship between ECM compositional changes and hemodynamic alterations in a model of aortic stiffness.

In general, it is understood that arteries remodel in response to mechanical loading from altered flow and pressure patterns [2,3] even though in humans, aortic stiffness may precede arterial hypertension [4,5]. Vascular remodeling processes include both structural and material transformations. Structural remodeling involves VSMC, fibroblast, and endothelial cell reorganization, and enhanced matrix turnover and deposition and furthermore is dependent upon the integrity of the adaptive immune system [6]. Material remodeling involves post-translational modifications, including two forms of cross-linking. The non-enzymatic advanced glycation end products (AGE) are cross-links that occur spontaneously [7], whereas enzymatic family of lysyl oxidases (LOX; EC 1.4.3.13, protein-lysine 6-oxidase), form co-valent vascular matrix cross-links [8] under the control of numerous growth factors and cytokines [9]. The functional enzymatic activity of LOX is the post-translational oxidative deamination of the ε-amino group of lysine and hydroxylysine ECM residues followed by the spontaneous condensation to di-valent and under select conditions tri-valent cross-links [10]. While the natural formation of vascular ECM cross-linking stabilizes the fibrous ECM proteins in a beneficial manner, over-abundance of collagen cross-linking renders collagen resistant to proteinase degradation [11]. There are five members of the LO family: lysyl oxidase (LOX) and lysyl oxidase-like 1–4 (LOXL 1–4) [12]. In the aorta LOXL1 appears to be important for the ECM integrity [13] and evidence suggests that the LOX isoform is essential for normal vascular development and function [14]. In addition to collagen, elastin is a substrate for lysyl oxidase mediated cross-linking through the formation multivalent cross-links which results in conformational constraints [15]. We propose that the overabundance of LOX mediated vascular cross-linking contributes to vascular stiffness.

A key issue is that a stiffer aorta alters the blood flow patterns to the kidney, brain, and cardiovascular system resulting in pathology [16,17]. With increased arterial stiffness there is increased pulse wave velocity (PWV) with a correspondingly early return of the reflection wave (RW), which together augment systolic pressure, increase wall stress, and alter endothelial cell function [5,18,19]. According to the Windkessel model, the aorta acts as a hydraulic capacitor that reduces systolic flow and increases diastolic flow in the distal aorta, thus measured as diastolic flow fraction (DFF). The reduction of DFF, secondary to increased aortic stiffness, adds to the cardiac hemodynamic load and energy utilization, and negatively affects blood flow patterns in the downstream arteries and organs [20].

The contribution of LOX mediated matrix cross-linking associated with vascular stiffness and function requires further investigation. The central objective of this study is to show, in an angiotensin II (Ang II) model of aortic stiffness, that inhibition of LOX mediated vascular ECM cross-linking with β-aminopropionitrile (BAPN) reduces the Ang II induced aortic stiffness and thereby central aberrant aortic flow patterns. We describe the Ang II mediated functional effects in vivo of aortic stiffness with PWV, Young’s modulus, RW time, and DFF values, all derived from flow wave Doppler recordings. Structural effects of remodeling were characterized with perfusion fixed histological specimen using morphometry and collagen bioassays. The material changes were assessed with bench collagen assays. We find that LOX inhibition suppresses the Ang II-mediated increase in LOX enzymatic activity and material based aortic stiffness without affecting structural remodeling.

## Materials and Methods

### Mice and study design

Three-month-old male C57BL/6J mice were obtained from Jackson Laboratories (Bar Harbor, ME, USA). This study was approved by the University of Arizona Animal Care Committee and conforms to the Guide for the Care and Use of Laboratory Animals published by the US National Institutes of Health (NIH Publication No. 85–23, revised 1996). All mice were randomly divided into placebo and three treatment groups.

A two by two study design was used to determine the effects of Ang II and BAPN on vascular stiffness. The BAPN (β-aminopropionitrile, Sigma-Aldrich) groups were pre-treated for 1 week prior to any test conducted. BAPN was dissolved into drinking water at a dosage of 1.5g/L *ad libitum*. Ang II was administered with a subcutaneous Alzet micro-osmotic pump (Durect Corporation, Cupertino, CA) releasing the Ang II receptor type I (AT1) agonist [Val^5^]Ang II (Sigma) at a rate of 490ng/kg/min. Control groups also received the pumps filled with vehicle (PBS). On Day 14, the mice were sacrificed for descriptive analyses.

### Blood Pressure Measurements

All mice were trained with the tail cuff system and data were recorded for Days 0 and 14. Blood pressure values were measured with the tail cuff system while the mice were placed on a heated platform (Hatteras Instruments, Cary, North Carolina). Blood pressure values recorded were from an average of ten consecutive measurements.

### ECHO/Doppler

Pulse wave velocity (PWV), reflection wave (RW) transit time, and diastolic flow fraction (DFF) were calculated from noninvasive ECHO Doppler measurements acquired with the Vevo 770 High Resolution Imaging System (VisualSonics, Toronto, Canada) version 2.3.0. using the model 707B scan head. Measurements were taken in the supine position with a heated platform while the mice were anesthetized with 1.3% isoflurane with continuous electrocardiogram (EKG) monitoring, with heart rates (HR) ranging from 325–375 beats per minute. The pulse wave velocity was calculated based on the time difference between the arrival of the leading edge of the systolic pulse wave at the innominate artery and at a position just superior to the renal artery. The wall stiffness, Eh, was derived from the Moens-Korteweg equation for pulse wave velocity, where E is the Young’s modulus and h is the wall thickness. The effective Young’s modulus, E, was then deduced using data on wall thickness. The methods for measuring and computing the PWV, DFF, and RW transit time are described in the Supplement.

### Determination of LOX Activity

LOX activity was measured by a fluorescent assay modified from the report of Palamakumbura and Trackman [21]. Briefly, 25mg of whole aorta tissue was homogenized in 300μL of Cell lytic (Sigma) at -80°C and reconstituted to 1mL with LOX buffer (1.2 mol/L urea, 50mmol/L sodium borate [pH = 8.2]) then centrifuged at 12,000 g for 10 minutes. Supernatant was then used for LOX enzymatic activity through the production of H_2_O_2_ and detected with fluorescent resorufin at wavelength 563 and 587nm. BAPN was used in one set of paired samples to establish that the production of H_2_O_2_ was a specific product of lysyl oxidase enzymatic activity.

### Perfusion Fixation and Histological Staining

Mice were anti-coagulated with a subcutaneous injection of 100 μl 1,000 USP units/mL heparin sodium prior to sacrifice. Thirty mL of saline was infused into the left ventricle and out of the right atrium to remove vascular blood, followed by in situ fixation with the administration of 2:1 3% glutaldehyde:1% formaldehyde, to prevent fixation contraction, for 15 minutes at a constant perfusion pressure of 40 mmHg. Once fixed, the aortic tissue was carefully removed to preserve the vascular architecture. The tissues were paraffin embedded, cut at 5 μm, and stained with H&E, Masson’s Trichrome, Verhoeff’s Van Gieson (VVG) elastin stain and Picrosirius red (PSR) collagen stain. Image analysis was performed using NIH Image-J software to quantify aortic diameter and the tissue collagen related to the Ang II and BAPN treatments.

### Determination of Hydroxyproline and collagen cross-linking

Hydroxyproline and cross-linking assays were performed as previously described by Yu et al. [9] Briefly, total aorta hydroxyproline measurements were compared to a standard colorimetric curve of trans-hydroxyproline (sigma). The data was expressed in micrograms of collagen per milligram of aorta dry weight, assuming collagen contains an average of 13.5% hydroxyproline. Cyanogen bromide digestion was used to determine collagen cross-linking. Cross-linking percentage was expressed as total hydroxyproline to cyanogen bromide-soluble hydroxyproline according to Woodiwiss et al [22].

### Statistics

ANOVA with multi-comparison procedures was used to test the difference among the defined groups with SPSS version 11.5. Values obtained from treatment groups were compared with control values using Student’s t-test. Comparable non-parametric tests (Kruskal-Wallis and the rank sum test) were substituted when test for normality and equal variance failed. All data are reported as means ± SEM. Data are from the vascular stiffness study whose authors may be contacted at the University of Arizona, Saver Heart Center, Tucson, Arizona 85724. A complete dataset for this report is posted at DOI:10.5061/dryad.65525.

## Results

### Vascular effect of BAPN on Ang II-induced arterial hypertension

**Fig 1:**
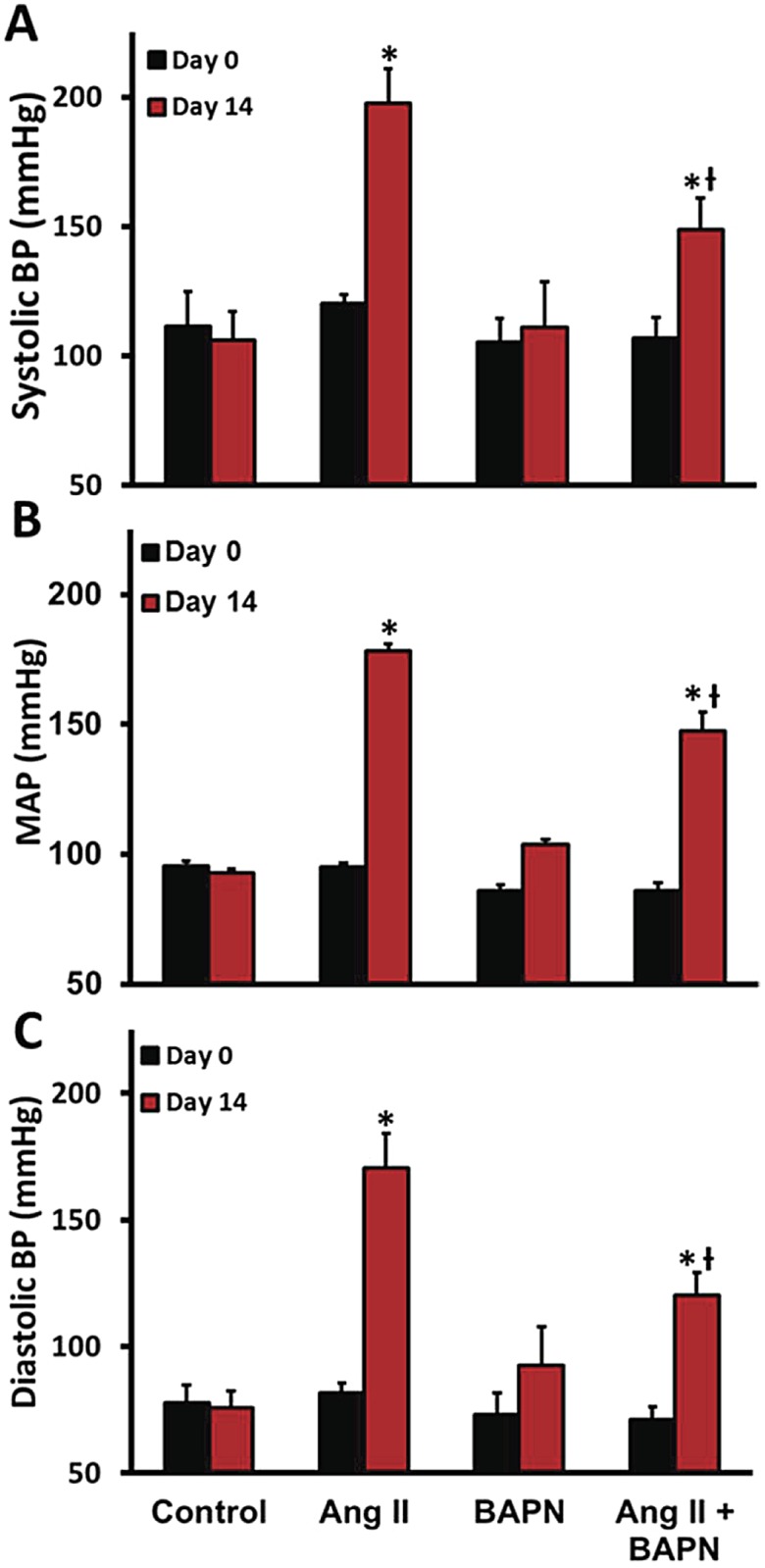
Vascular and cardiac effect of BAPN on Ang II induced arterial hypertension. To assess the effects of Ang II and LOX inhibition on vascular function, we first analyzed the systemic arterial blood pressure measured with tail cuff. **A**, The systolic arterial blood pressure, **B**,The mean arterial blood pressure. **C**, Diastolic arterial blood pressure. n = 6. *P<0.05 compared to control, ^Ɨ^P<0.05 compared with BAPN group, ^#^P<0.05 compared with Ang II group at their respective day.

Fig 1 demonstrates that 14 days of Ang II alone increases arterial blood pressure by greater than 60%. BAPN was administered for 7 days prior to Ang II and continued for the 14 days concurrent with the Ang II infusion. Fig 1B shows that administration of BAPN alone lowered the Day 0 mean arterial blood pressure (MAP) by 10% from 95 ± 2 to 86 ± 2 mmHg (P = 0.02). When the BAPN was given with Ang II for 14 days it lowered the MAP 18% from 178 ± 3 to 147 ± 7 mmHg (P<0.001). When comparing the systolic pressures (Fig 1A) with the diasolic pressures (Fig 1C), Ang II alone decreased the pulse pressure from 38 ± 2 to 27 ± 4 mmHg (P = 0.002), though the addition of BAPN to the Ang II did not alter the pulse pressure; 35± 5 versus 32 ± 1 mmHg (NS) when comparing Day 0 with 14. Neither Ang II infusion nor BAPN administration affected the heart rate.

### Aortic flow characteristics

**Fig 2:**
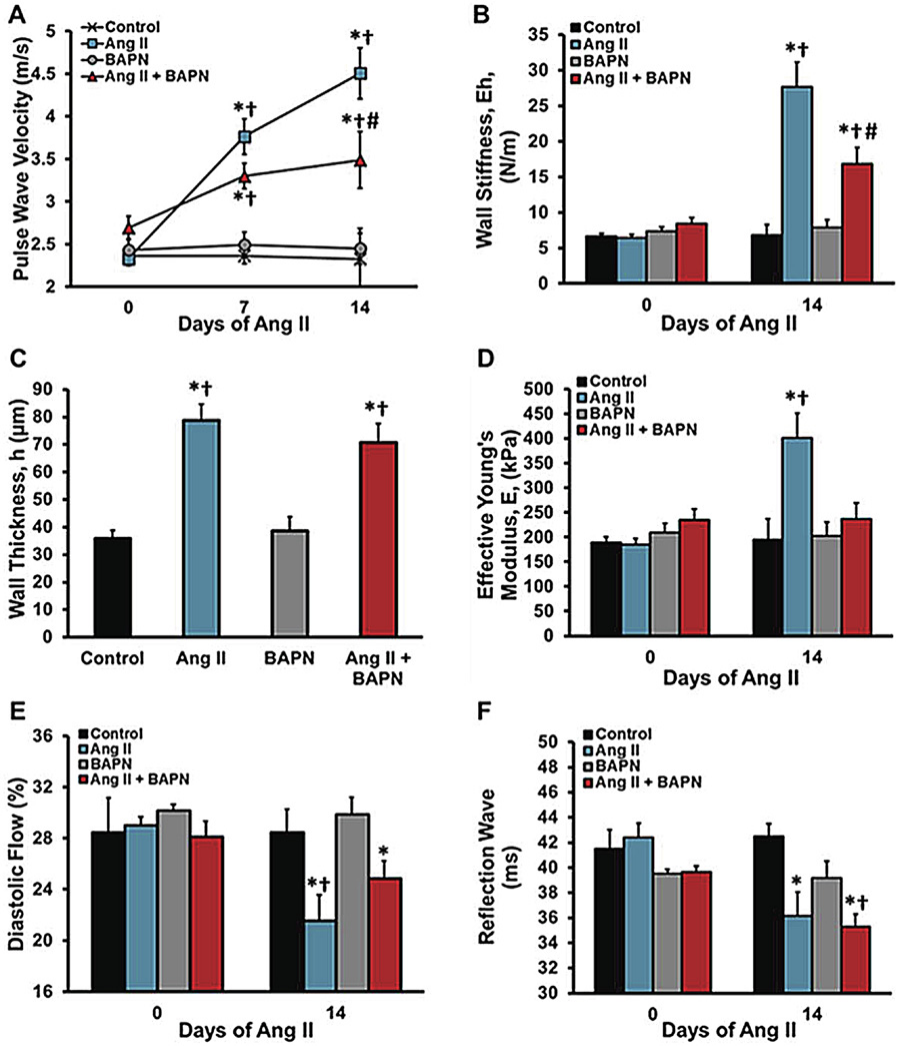
Aortic flow and stiffness characteristics. The flow characteristics of the lower thoracic aorta, superior to the renal bifurcation, were analyzed with ECHO/Doppler. **A**, Pulse wave velocity was used to determine vascular stiffness. **B**, Effective wall stiffness (Eh), was deduced from measuring PWV. **C**, Wall thickness (h), was measured from perfusion fixed aortas at day 14, at the same location of the PWV readings. **D**, The effective Young’s modulus (E), readings were made weekly. **E**, The diastolic flow percentages was calculated comparing the systolic and diastolic VTI’s. **F**, The reflected wave transit time was measured at the innominate artery with flow Doppler. n = 6. *P<0.05 compared to control, ^Ɨ^P<0.05 compared with BAPN group, ^#^P<0.05 compared with Ang II group at their respective day.

The central question of this investigation is to determine whether LOX enzymatic activity affects vascular mechanics. All data shown in Fig 2 were derived from flow wave Doppler recordings. Aortic stiffness was analyzed using pulse wave velocity (PWV) based on the transit time and distance between the innominate artery and the abdominal aorta above the renal arteries. Ang II increased PWV while BAPN alone had no effect compared with the control group. Fig 2A shows a 33% reduction in PWV in the BAPN + Ang II group when compared to the Ang II only group. The wall stiffness, Eh, computed from PWV (Fig 2B) demonstrated that Ang II caused a 4.5-fold increase in wall stiffness whereas co-administration of BAPN with Ang II led to a 2.5-fold increase (P<0.05). Fig 2C shows total wall thickness, h, in the abdominal aorta. Both Ang II alone and Ang II with BAPN caused a 2-fold increase in intimal-medial-adventitial wall thickness. The effective Young’s modulus, E, was estimated by dividing the wall stiffness, Eh, by the measured wall thickness, h. Most importantly, Fig 2D demonstrates that Ang II alone caused a 2-fold increase in effective Young’s modulus which was inhibited by BAPN.

To analyze the effects of BAPN on the diastolic flow fraction (DFF) of the abdominal aorta, the velocity-time-integral was measured separately for the systolic and diastolic flow phases and computed individually into systolic and diastolic flow per minute. Fig 2E shows that Ang II alone decreased the DFF from 29.0± 0.7 to 21.5 ± 0.6% (P<0.001) and thus there was a correspondingly increase in systolic flow fraction from 8.7 to 11.7 mL/min (P = 0.02). BAPN attenuated the decrease in diastolic flow fraction mediated by Ang II to 24.8± 1.6%, compared with Ang II alone (P<0.05). Since the majority of aortic capacitance resides in the thoracic region, these data show that Ang II mediated LOX enzymatic function significantly affects this regional capacitance function and thus down-stream aortic flow characteristics. The third in vivo analysis of vascular stiffness was the reflected wave (RW) transit time relative to the EKG-R wave in the innominate artery (Fig 2F). To validate an estimated RW transit time, we first measured the transit time from the innominate artery to the iliac arteries which was 21.0 ± 0.5 ms in control mice. Assuming that the iliac arteries are a source of reflected waves, the reflected wave should be detected at the innominate artery approximately twice the forward transit time; that is 42 ms. Our measured RW transit time relative to the R wave in the innominate artery is 42.4 ± 1.3 ms. As shown in Fig 2F, the RW transit time was reduced from 42.4 ± 1.1 to 36.1 ± 1.1 (P = 0.029) with Ang II and no change with concurrent BAPN administration. These data suggest that Ang II increases PWV, and decreases Young’s modulus, DFF and RW time, and LOX inhibition reduces vascular stiffness as measured with PWV and DFF but does not affect RW transit time.

### Cessation of Ang II infusion

**Fig 3:**
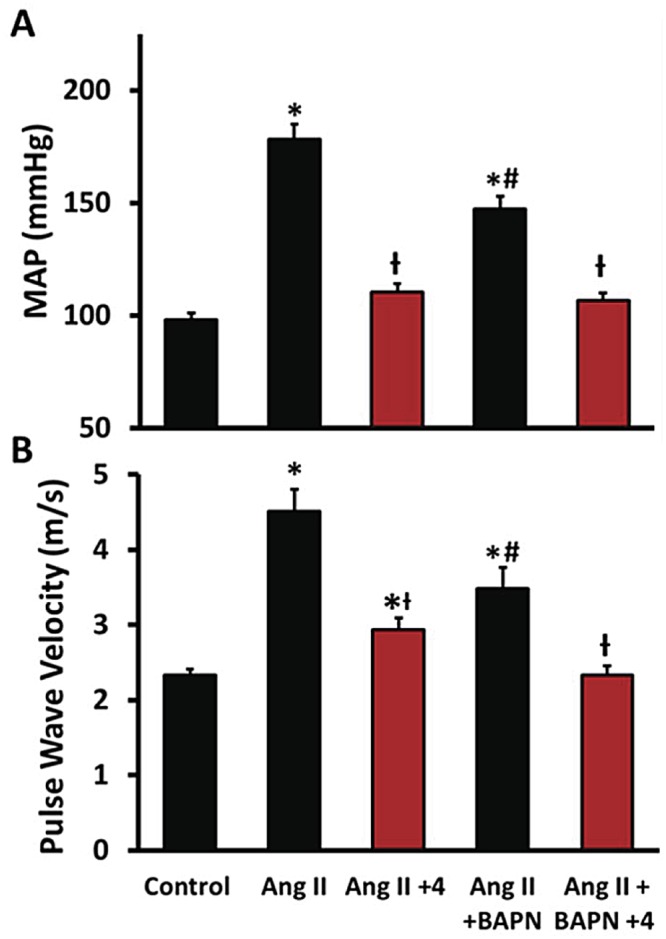
Cessation of Ang II infusion. Hypertension contributes to vascular stiffness together with structural and material remodeling. Therefore the Alzet pumps were removed to determine the contribution of each to the total vascular stiffness. **A**, The Alzet pumps were removed on Day 14 and the blood pressure returned to control values by day + 4. **B**, The pulse wave velocity measured with and without Ang II infusion on days 14 and + 4 days after pump removal. n = 6. *P<0.05 compared to control, ^Ɨ^P<0.05 compared with Ang II group, ^#^P<0.05 compared with Ang II + BAPN + 4 group.

Hypertension alone can contribute to the measured increase in vascular stiffness due to the alignment and engagement of collagen fibers that occurs with increased circumferential strain and also due to increased VSMC tone. To distinguish these effects from changes in material properties, we removed the Ang II infusion pumps from the Day 14 mice and four days later assessed the PWV. Removal of the Ang II pumps resulted in restoration of the arterial blood pressures to control values (Fig 3A). More specially, Fig 3B shows that the PWV increased from 2.33 ± 0.3 to 4.51 m/s (P<0.0001) with chronic Ang II infusion and decreased to 2.93 ± 0.16 m/s (-65%, P<0.002) with pump withdrawal. We infer that the reduction from 4.51 m/s to 2.93 m/s after Ang II pump withdrawal is due to the vasoactive effects of Ang II and/or effects of increased strain, and the difference, a 35% residual, between the control PWV of 2.33 m/s and the value of 2.93 m/s after Ang II pump withdrawal is due to ECM remodeling. In support of this conclusion, BAPN inhibition of ECM crosslinking resulted in a lowered Ang II PWV and Ang II pump withdrawal in the Ang II + BAPN group returned the PWV by 99% to control values of 2.34 ± 0.0.08 m/s. Since there were no differences between the collagen and elastin concentrations when comparing the Ang II group with the Ang II plus BAPN group; these data strongly suggest that Ang II administration alone induces both increased vascular strain and remodeling that includes those enzymatic effects of LOX whereas LOX does not inhibit morphological remodeling but inhibits functional or effectual remodeling.

### Vascular morphology

**Fig 4:**
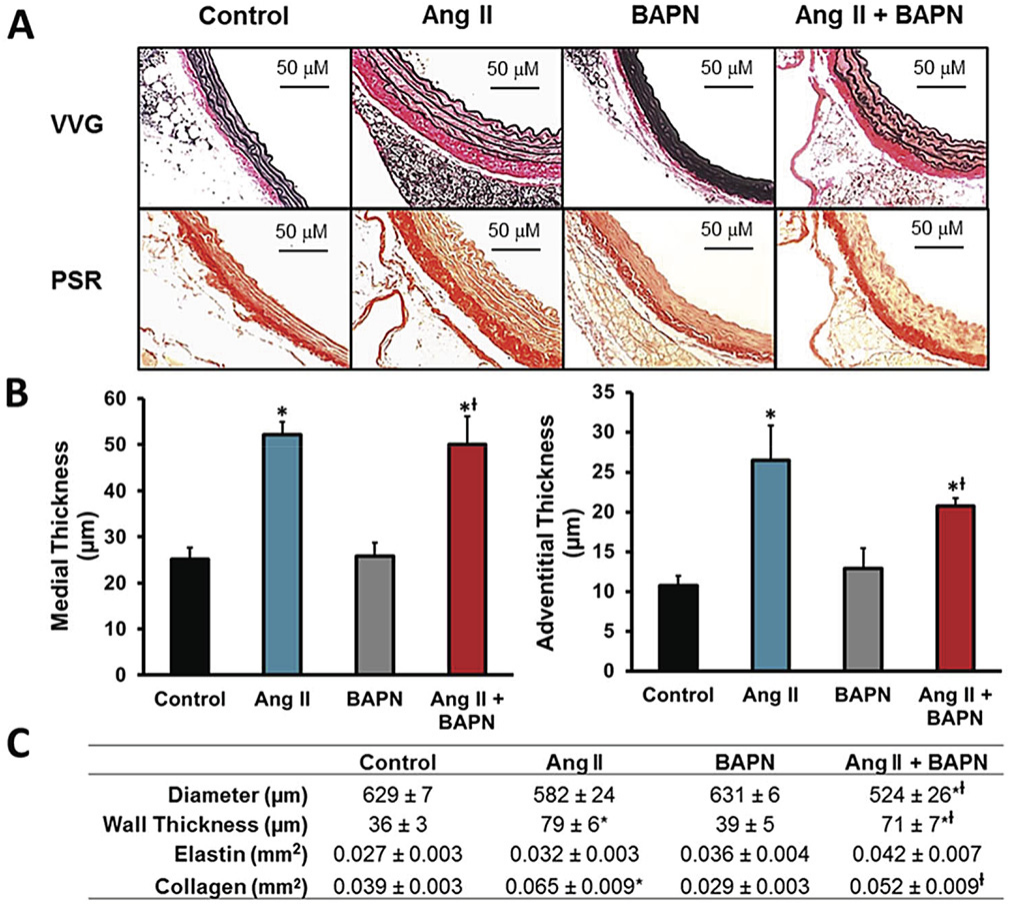
Vascular morphology. Aortic histomorphometry was performed with the elastin specific stain Verhoeff’s Van Gieson (VVG) and the collagen specific stain picrosirius red (PSR). **A**, Representative histological sections are presented according to treatment. **B**, The medial thickness and adventitial thickness were analyzed to describe the aortic morphological changes in response to the treatments. **C**, The aortic structural dimensions as measured with VVG and PSR stains. **D**, The systolic luminal diameter were measured with the M-mode ECHO, collagen content was quantified with PSR histological staining, and elastin content was measured with VVG stain. n = 6. *P<0.05 compared to control, ^Ɨ^P<0.05 compared with BAPN group.

Based on the measured effects of LOX inhibition on vascular function, we assessed the effect of Ang II with and without BAPN on vascular structural remodeling. On Day 14 the aortas were perfusion fixed *in situ* at a pressure of 40 mmHg and then the upper abdominal aortas were harvested. Fig 4A shows altered medial and adventitial thicknesses and specific stains for elastin and collagen. Verhoeff’s Van Gieson (VVG) staining of elastin (black), and Picrosirius red (PSR) staining (red) demonstrate the regional distribution of elastin and collagen in the artery, respectively. The groups receiving BAPN had punctated collagen distribution in the medial layer. Medial thickness increased in both groups receiving Ang II and not affected by BAPN (Fig 4B) BAPN also failed to reduce Ang II-induced adventitial hyperplasia (Fig 4B). Fig 4C defines the vessel dimensions and levels of elastin and collagen using morphometric analysis. Ang II increased vascular collagen content by 67% (P<0.01) whereas BAPN caused a 26% reduction of vascular collagen (P = 0.08) compared with control. Fig 4C also shows that the combination of Ang II + BAPN led to significantly more total collagen than the control and BAPN groups, but less than the Ang II alone. The systolic aortic diameter measured from an M-mode upper aortic ECHO was significantly reduced in the Ang II plus BAPN groups compared with the control group (P<0.02). It was noted that 30% of the Ang II plus BAPN mice had aortic aneurysms; these mice were not used in the data presented since the aneurysms involved an anatomical region that was critical to our stiffness analysis. Taken together, Ang II causes significant changes morphological properties of the aortic vasculature which are not reversed by LOX inhibition.

### LOX enzymatic effects

**Fig 5:**
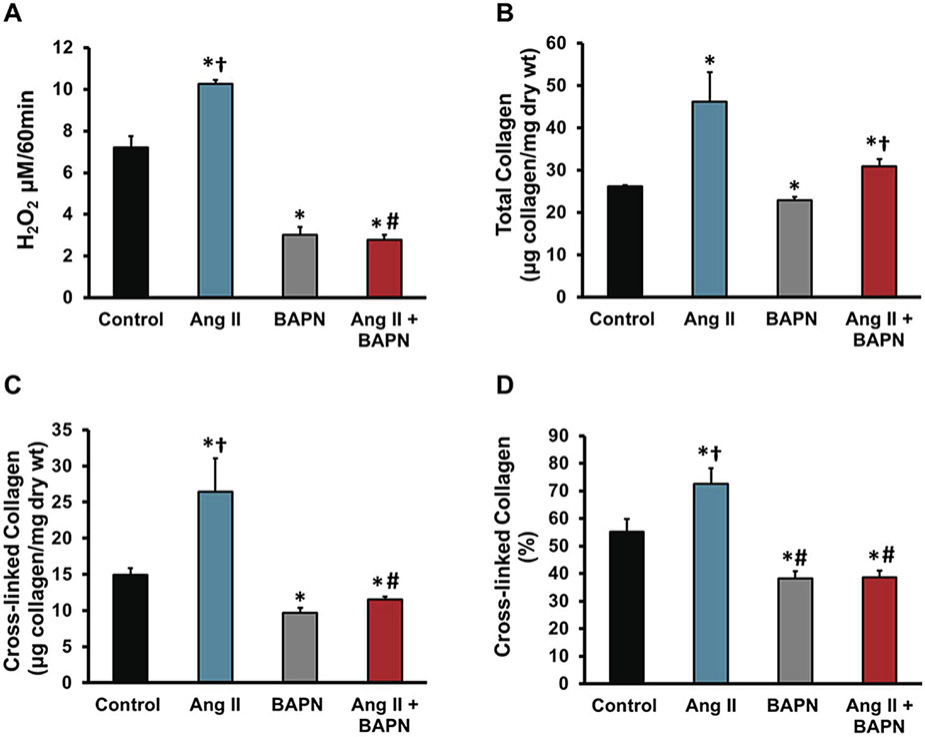
Effect of Ang II and BAPN on LOX, collagen, and cross-linking in aortas. The effect of LOX is not confined to the crosslinking of collagen and elastin and therefore we examined the effect of LOX on gene expression of other key ECM proteins and enzymes. **A**, Lysyl-oxidase enzymatic activity measured from the lower thoracic aorta represented by the production of H_2_O_2_ and detected by Amplex red oxidation. **B**, The total collagen content using hydroxyproline concentrations assuming collagen contains 13.5% hydroxyproline. **C**, Cross-linked collagen, using cyanogen bromide as digestion. **D**, Percent of collagen cross-linking. n = 6. *P<0.05 compared to control, ^Ɨ^P<0.05 compared with BAPN group, ^#^P<0.05 compared with Ang II group at their respective day.

We have shown the hemodynamic, histological, and vascular mechanical changes that occur with BAPN treatment. To directly determine the effect of BAPN on LOX enzymatic activity, LOX enzymatic activity assay was measured in aortic tissue after 21 days of BAPN treatment. The assay of LOX activity was the spectrophotometric detection of the byproduct of LOX enzymatic activity, hydrogen peroxide. Fig 5A shows that Ang II increases the enzymatic activity of LOX in the aorta (P<0.01) and that BAPN significantly reduces the enzymatic activity with or without Ang II stimulation (P<0.01). Furthermore we indirectly measured LOX cross-linking, by comparing total vs soluble collagen using hydroxyproline assay (Fig 5B–5D). Total collagen measurements in Fig 5B show an increase in total vascular collagen in Ang II and Ang II plus BAPN by 44% and 14% respectively, whereas BAPN alone significantly decreased collagen content by 15%. Insoluble or cross-linked collagen is shown in Fig 5C. Cyanogen bromide digestion was used to determine cross-linking, showed significant decreases in BAPN administration regardless of the Ang II stimulation (P<0.02), and a 44% increase in Ang II alone (P<0.05). Comparing the soluble to total collagen of the samples, we were able to show the indirect measurement of collagen cross-linking as a percentage (Fig 5D). These spectrophotometric assays show BAPN significantly reduced vascular collagen content in addition with cross-linked collagen ratios.

## Discussion

### Overview of findings

The ability of the arteries to remodel in response to flow-dependent and -independent stimuli is central to most arterial diseases. We hypothesized that ECM collagen cross-links contribute to arterial remodeling, and thereby influence aortic stiffness and hemodynamic characteristics. The goal of this study was to modulate the material properties of the aorta mediated by the cross-linking enzyme, lysyl oxidase (LOX), with Ang II and the LOX inhibitor, BAPN, and then to assess the *in vivo* aortic stiffness. The functional parameters for aortic stiffness included the clinical standard, pulse wave velocity (PWV); diastolic flow fraction (DFF); and reflection wave (RW) transit time, which were computed from Doppler flow measurements. We found that chronic Ang II infusion increased PWV, and reduced DFF and RW transit time. Importantly, these parameters indicate flow patterns that are clinically associated with injury to major organ systems [5,18,19]. To determine what proportion of the measured vascular stiffness is caused by the vascular strain secondary to hypertension, we abruptly terminated the Ang II infusion. With normalized arterial blood pressure, we found a residual vascular stiffness representing effects related to material and structural remodeling. Furthermore, LOX inhibition in Ang II treated mice completely blocked the increase in effective Young’s modulus and partially normalized PWV and DFF while having no effect on RW transit time. Since LOX has been primarily associated with cross-linking of the vascular ECM proteins, these results imply that blocking the formation of ECM cross-linking leads to normalization of the material properties as described by the effective Young’s modulus. Taken together, these results show that Ang II increases vascular stiffness through a combination of increased wall thickness, increased strain resulting from hypertension, and changes in the intrinsic material properties of the wall mediated by LOX enzymatic activity.

### Vascular Function

Our *in vivo* measurements of aortic stiffness were computed from flow wave Doppler. The PWV was determined with the flow wave Doppler transit times, measured from the EKG-R wave at two anatomical sites; the innominate artery and the abdominal aorta superior to the renal artery bifurcations. The PWV was markedly increased with Ang II infusion suggesting vascular stiffness. The technique using flow wave Doppler was previously described [23] and provided a correlation (r = 0.83, P<0.0001) with the standard pulse pressure method using applanation tonometry [24]. Due to its elastic compliance, the aorta stores a portion of the cardiac systolic stroke volume and discharges this volume during diastole (the Windkessel effect). The DFF represents diastolic flow as a fraction of total flow. DFF was measured at the abdominal aortic site. Ang II significantly reduced the DFF and suggesting increased proximal aortic stiffness. Lastly, an increase in arterial stiffness decreases the RW transient time at the innominate artery and is predictive of vascular pathology [17]. We found that the RW transient time was shorter in the Ang II mice. A major benefit of our use of flow wave Doppler analysis is that it is non-invasive, thus permitting a longitudinal analysis of vascular stiffness in each animal.

The most striking finding was that BAPN partially inhibited Ang II induced vascular wall stiffening but completely inhibited the increase in effective Young’s modulus. Since Young’s modulus describes the material properties, this observation suggests that vascular stiffness is dependent upon LOX mediated cross-linking. The mechanical properties of the arterial vasculature have been shown to be directly related to the comparative concentrations of vascular ECM fibrillar proteins and the levels of ECM protein cross-linking [10]. To strengthen our observation, we found that BAPN had minimal effects on structural changes caused by Ang II, moderate effects of PWV and DFF, and no effect on the RW Transient time. Other reported vascular effects of BAPN have been to reduce blood pressure in a deoxycorticosterone acetate (DOCA) hypertensive model [25], and *ex-vivo* stiffness in sectioned vascular rings [8,26].

### Material, Structural, and Functional

We investigated arterial vascular stiffness in terms of material, structural, and blood pressure. We have shown that Ang II increases the material properties through increased vascular enzymatic activity of LOX, collagen content, and cross-linked collagen which were inhibited with BAPN. BAPN inhibits all isoforms of lysyl oxidase however the differential vascular expression of the LOX and Lysyl oxidase-like (LOXL-1) isoforms have been shown to be critical in age dependent aortic vascular function [13,27]. Taken from the above reports, we suggest that our BAPN treatments are affecting primarily the adult isoform, LOXL-1, in the aorta. Ang II also caused structural increases in medial and adventitial thicknesses which were not affected by LOX inhibition. It has been suggested that less than ten percent of collagen fibers are engaged at physiological pressures [28], whereas with biaxial loading, as observed with hypertension, the vessel becomes stiffer due to a sequential realignment and engagement of the medial and then adventitial collagen fibers [29]. Therefore in our study the measured stiffness in the arterial hypertensive state is partially due a right-ward shift of the stress/strain relationship resulting in the recruitment of collagen fibers. To directly separate the effect of blood pressure and remodeling on vascular stiffness, we withdrew the Ang II infusion pumps on Day 14. Discontinuation of Ang II infusion resulted in an immediate decrease in blood pressure to control values and a 70% decrease in PWV measurement. Therefore, the remaining residual stiffness of 30% represents material and structural remodeling. Our results suggest, in this Ang II model, that the majority of vascular stiffness may be due to the hypertensive state. Moreover with pump withdrawal in the Ang II/BAPN group showed a complete return of PWV control levels, suggesting that the cross-linked material properties contribute substantially to the residual stiffness.

Classically, it has been proposed that the vascular remodeling response alters the structural and material properties in order to restore normal biomechanical parameters [2]. Others have proposed that the overall vascular stiffness of large arteries includes both the intrinsic mechanical properties of the VSMC in addition with the material and structural ECM remodeling [30] which is consistent with our observations. To expand this concept further, the VSMC contribution to vascular stiffness is relatively dependent upon the altered ECM environment and connectivity [31]. There appears to be a sequential transition of vascular stiffness including VSMC tone and collagen realignment responses to support short-term needs and an adaptive process including cellular proliferation, matrix production and maturation, and connectivity of the contractile apparatus for more chronic conditions. Therefore the arterial vascular adapts in a prescribed order to achieve “mechanical homeostasis” in response to the duration and types of stressors [32].

### Ang II mediated Vascular Remodeling

In this study we used the AT1 agonist [Val^5^]-angiotensin II. Ang II, through its AT1 receptor, is known to induce VSMC vasoconstriction in addition to induce collagen production by the VSMCs [33] and adventitial fibroblasts [34]. The understood pathway is activation of the AT1 receptor stimulates of TGF-β and its downstream mediator, connective tissue growth factor (CTGF) [35] leading to collagen accumulation. Yet it appears that LOX inhibition impairs Ang II-induced vascular remodeling and mechanics irrespective of collagen content. LOX may have other undefined targets on an array of processes that regulate structural and material remodeling. However our data suggest that LOX inhibition impairs Ang II-induced vascular remodeling and mechanics through decreased collagen content and maturation.

### Integration

Our *in vivo* investigation reveals that Ang II produces vascular stiffness by affecting the blood pressure through VSMC tone, ECM function through increased stress, and material and structural properties through remodeling. Since we have shown that Ang II induces LOX expression [6], the central aim of this report was to show that Ang II infusion induces vascular LOX enzymatic activity which is associated with vascular material stiffness through ECM cross-linking. This Ang II mediated cross-linking can be inhibited resulting in a significant decrease in vascular stiffness. The importance of a balanced LOX expression in the arterial vasculature is further supported by the observations that LOX inhibition can result in medial and elastic laminar degeneration resulting in aneurysm formation [36,37] and conversely stimulation of LOX-mediated cross-linking stabilizes aortic aneurysms [38]. Elevated LOX activity has been shown to induce atherosclerosis and inhibition has led to plaque instability as reviewed by Rodriguez [39]. Evidently, therefore, LOX is a very important enzyme for maintaining the vascular homeostasis of the ECM. A clinical implication of this work is that stiffening of the aorta due to enhanced LOX enzymatic function could be detrimental to the heart, large arteries, as well as the end-organs in hypertensive, and aged individuals.

### Study limitations

We have shown the *in vivo* effects of LOX on vascular stiffening and described this through the cross-linking of collagen. However elastin can potently form up to 4 times the number of lysyl oxidase mediated cross-links that may occur in a tetravalent form. The types of cross-links occurring in collagen proteins are known to be on C- and N-terminal with intra-collagen cross-links however elastin proteins requires a three-dimensional network needed during for fiber assembly [15]. It is speculated that the tertiary structure imposes conformational constraints that may differ from that of collagen. We used an established analytical method for collagen cross-linking quantification as a means to describe the effect of Ang II and LOX in vascular stiffening and did not assay the elastin due to the difficulty in analysis.

Aortic mechanical function *in vivo* is a function of both the large arteries and smaller resistance arteries. In this study we did not directly measure smaller arterial function, although changes in their properties are reflected in the reported arterial blood pressures.

## Supporting Information

S1 Fig Defining the parameters for computation of PWV, DFF, and RW transit time. Transit times T1 and T2 were determined by the time from the peak of the EKG-R wave and the first pixel increase on their respective flow tracing. The distance between the innominate artery and the abdominal aorta just superior to the renal bifurcation was and T2 minus T1 was used to determine PWV. RW transit time was measured from the peak of the EKG-R wave and the nadir of the innominate artery flow Doppler. The DFF was computed by the area of diastole flow (DF) compared with the area of systolic flow (SF).
(TIF)

S2 Fig Representative Flow Wave Doppler tracings. The red line represents the peak EKG-R wave. The yellow lines are the transit times from the R-wave and the first pixel increase in the flow tracing. The white line represents the nadir of the flow tracing in the innominate artery for the measurement of the RW transit time. The dotted blue line is the diastolic flow in the control aorta proximal to the renal arteries.
(TIF)

S1 Methods Methods for computation of PWV, DFF, and RW transit time. (DOCX)

## References

[1] Saphirstein RJ, Morgan KG (2014) The contribution of vascular smooth muscle to aortic stiffness across length scales. Microcirculation 21: 201–207. 10.1111/micc.12101 24635219 PMC8588963

[2] Humphrey JD (2008) Mechanisms of arterial remodeling in hypertension: coupled roles of wall shear and intramural stress. Hypertension 52: 195–200. 10.1161/HYPERTENSIONAHA.107.103440 18541735 PMC2753501

[3] Mitchell GF, Lacourciere Y, Ouellet JP, Izzo JL Jr., Neutel J, Kerwin LJ, et al (2003) Determinants of elevated pulse pressure in middle-aged and older subjects with uncomplicated systolic hypertension: the role of proximal aortic diameter and the aortic pressure-flow relationship. Circulation 108: 1592–1598. 12975261 10.1161/01.CIR.0000093435.04334.1F

[4] Dernellis J, Panaretou M (2005) Aortic stiffness is an independent predictor of progression to hypertension in nonhypertensive subjects. Hypertension 45: 426–431. 15710784 10.1161/01.HYP.0000157818.58878.93

[5] Kaess BM, Rong J, Larson MG, Hamburg NM, Vita JA, Levy D, et al (2012) Aortic stiffness, blood pressure progression, and incident hypertension. JAMA 308: 875–881.. 10.1001/2012.jama.10503 22948697 PMC3594687

[6] Majeed B, Tawinwung S, Eberson LS, Secomb TW, Larmonier N, Larson DF (2014) Interleukin-2/Anti-Interleukin-2 Immune Complex Expands Regulatory T Cells and Reduces Angiotensin II-Induced Aortic Stiffening. Int J Hypertens 2014: 126365 10.1155/2014/126365 25258681 PMC4167213

[7] Aronson D (2004) Pharmacological prevention of cardiovascular aging—targeting the Maillard reaction. Br J Pharmacol 142: 1055–1058. 15237100 10.1038/sj.bjp.0705832 PMC1575180

[8] Bruel A, Ortoft G, Oxlund H (1998) Inhibition of cross-links in collagen is associated with reduced stiffness of the aorta in young rats. Atherosclerosis 140: 135–145. 9733224 10.1016/s0021-9150(98)00130-0

[9] Yu Q, Vazquez R, Zabadi S, Watson RR, Larson DF (2010) T-lymphocytes mediate left ventricular fibrillar collagen cross-linking and diastolic dysfunction in mice. Matrix Biol 29: 511–518. 10.1016/j.matbio.2010.06.003 20600894 PMC2939274

[10] Reiser K, McCormick RJ, Rucker RB (1992) Enzymatic and nonenzymatic cross-linking of collagen and elastin. FASEB J 6: 2439–2449. 1348714 10.1096/fasebj.6.7.1348714

[11] van der Slot-Verhoeven AJ, van Dura EA, Attema J, Blauw B, DeGroot J, Huizinga TW, et al (2005) The type of collagen cross-link determines the reversibility of experimental skin fibrosis. Biochim Biophys Acta 1740: 60–67. 15878742 10.1016/j.bbadis.2005.02.007

[12] Csiszar K (2001) Lysyl oxidases: a novel multifunctional amine oxidase family. Prog Nucleic Acid Res Mol Biol 70: 1–32. 11642359 10.1016/s0079-6603(01)70012-8

[13] Behmoaras J, Slove S, Seve S, Vranckx R, Sommer P, Jacob MP (2008) Differential expression of lysyl oxidases LOXL1 and LOX during growth and aging suggests specific roles in elastin and collagen fiber remodeling in rat aorta. Rejuvenation Res 11: 883–889. 10.1089/rej.2008.0760 18803461

[14] Maki JM, Rasanen J, Tikkanen H, Sormunen R, Makikallio K, Kivirikko KI, et al (2002) Inactivation of the lysyl oxidase gene Lox leads to aortic aneurysms, cardiovascular dysfunction, and perinatal death in mice. Circulation 106: 2503–2509. 12417550 10.1161/01.cir.0000038109.84500.1e

[15] Brown-Augsburger P, Tisdale C, Broekelmann T, Sloan C, Mecham RP (1995) Identification of an elastin cross-linking domain that joins three peptide chains. Possible role in nucleated assembly. J Biol Chem 270: 17778–17783. 7629078 10.1074/jbc.270.30.17778

[16] Hametner B, Wassertheurer S, Hughes AD, Parker KH, Weber T, Eber B (2014) Reservoir and excess pressures predict cardiovascular events in high-risk patients. Int J Cardiol 171: 31–36. 10.1016/j.ijcard.2013.11.039 24315153

[17] Weber T, Wassertheurer S, Rammer M, Haiden A, Hametner B, Eber B (2012) Wave reflections, assessed with a novel method for pulse wave separation, are associated with end-organ damage and clinical outcomes. Hypertension 60: 534–541. 10.1161/HYPERTENSIONAHA.112.194571 22585948

[18] Gulan U, Luthi B, Holzner M, Liberzon A, Tsinober A, Kinzelbach W (2014) Experimental investigation of the influence of the aortic stiffness on hemodynamics in the ascending aorta. IEEE J Biomed Health Inform. 10 1109. 10.1109/JBHI.2014.2322934 24833608

[19] Haidet GC, Wennberg PW, Finkelstein SM, Morgan DJ (1996) Effects of aging per se on arterial stiffness: systemic and regional compliance in beagles. Am Heart J 132: 319–327. 8701893 10.1016/s0002-8703(96)90428-7

[20] Guo X, Kassab GS (2003) Variation of mechanical properties along the length of the aorta in C57bl/6 mice. Am J Physiol Heart Circ Physiol 285: H2614–H2622. 14613915 10.1152/ajpheart.00567.2003

[21] Palamakumbura AH, Trackman PC (2002) A fluorometric assay for detection of lysyl oxidase enzyme activity in biological samples. Anal Biochem 300: 245–251. 11779117 10.1006/abio.2001.5464

[22] Woodiwiss AJ, Tsotetsi OJ, Sprott S, Lancaster EJ, Mela T, Chung ES, et al (2001) Reduction in myocardial collagen cross-linking parallels left ventricular dilatation in rat models of systolic chamber dysfunction. Circulation 103: 155–160. 11136701 10.1161/01.cir.103.1.155

[23] Fitch RM, Rutledge JC, Wang YX, Powers AF, Tseng JL, Clary T, et al (2006) Synergistic effect of angiotensin II and nitric oxide synthase inhibitor in increasing aortic stiffness in mice. Am J Physiol Heart Circ Physiol 290: H1190–H1198. 16272204 10.1152/ajpheart.00327.2005

[24] Jiang B, Liu B, McNeill KL, Chowienczyk PJ (2008) Measurement of pulse wave velocity using pulse wave Doppler ultrasound: comparison with arterial tonometry. Ultrasound Med Biol 34: 509–512. 18031922 10.1016/j.ultrasmedbio.2007.09.008

[25] Iwatsuki K, Cardinale GJ, Spector S, Udenfriend S (1977) Reduction of blood pressure and vascular collagen in hypertensive rats by beta-aminopropionitrile. Proc Natl Acad Sci U S A 74: 360–362. 264688 10.1073/pnas.74.1.360 PMC393260

[26] Zhang L, Pei YF, Wang L, Liao MF, Lu QS, Zhuang YF, et al (2012) Dramatic decrease of aortic longitudinal elastic strength in a rat model of aortic dissection. Ann Vasc Surg 26: 996–1001. 10.1016/j.avsg.2012.02.004 22819525

[27] Maki JM, Sormunen R, Lippo S, Kaarteenaho-Wiik R, Soininen R, Myllyharju J (2005) Lysyl oxidase is essential for normal development and function of the respiratory system and for the integrity of elastic and collagen fibers in various tissues. Am J Pathol 167: 927–936. 16192629 10.1016/S0002-9440(10)61183-2 PMC1603668

[28] Greenwald SE, Moore JE Jr., Rachev A, Kane TP, Meister JJ (1997) Experimental investigation of the distribution of residual strains in the artery wall. J Biomech Eng 119: 438–444. 9407283 10.1115/1.2798291

[29] Chow MJ, Turcotte R, Lin CP, Zhang Y (2014) Arterial extracellular matrix: a mechanobiological study of the contributions and interactions of elastin and collagen. Biophys J 106: 2684–2692. 10.1016/j.bpj.2014.05.014 24940786 PMC4070071

[30] Sehgel NL, Zhu Y, Sun Z, Trzeciakowski JP, Hong Z, Hunter WC, et al (2013) Increased vascular smooth muscle cell stiffness: a novel mechanism for aortic stiffness in hypertension. Am J Physiol Heart Circ Physiol 305: H1281–H1287. 10.1152/ajpheart.00232.2013 23709594 PMC3840243

[31] McDaniel DP, Shaw GA, Elliott JT, Bhadriraju K, Meuse C, Chung KH, et al (2007) The stiffness of collagen fibrils influences vascular smooth muscle cell phenotype. Biophys J 92: 1759–1769. 17158565 10.1529/biophysj.106.089003 PMC1796816

[32] Humphrey JD (2008) Vascular adaptation and mechanical homeostasis at tissue, cellular, and sub-cellular levels. Cell Biochem Biophys 50: 53–78. 10.1007/s12013-007-9002-3 18209957

[33] Ford CM, Li S, Pickering JG (1999) Angiotensin II stimulates collagen synthesis in human vascular smooth muscle cells. Involvement of the AT(1) receptor, transforming growth factor-beta, and tyrosine phosphorylation. Arterioscler Thromb Vasc Biol 19: 1843–1851. 10446062 10.1161/01.atv.19.8.1843

[34] Che ZQ, Gao PJ, Shen WL, Fan CL, Liu JJ, Zhu DL (2008) Angiotensin II-stimulated collagen synthesis in aortic adventitial fibroblasts is mediated by connective tissue growth factor. Hypertens Res 31: 1233–1240. 10.1291/hypres.31.1233 18716373

[35] Rodriguez-Vita J, Sanchez-Lopez E, Esteban V, Ruperez M, Egido J, Ruiz-Ortega M (2005) Angiotensin II activates the Smad pathway in vascular smooth muscle cells by a transforming growth factor-beta-independent mechanism. Circulation 111: 2509–2517. 15883213 10.1161/01.CIR.0000165133.84978.E2

[36] Kanematsu Y, Kanematsu M, Kurihara C, Tsou TL, Nuki Y, Liang EI, et al (2010) Pharmacologically induced thoracic and abdominal aortic aneurysms in mice. Hypertension 55: 1267–1274. 10.1161/HYPERTENSIONAHA.109.140558 20212272 PMC2859958

[37] Kurihara T, Shimizu-Hirota R, Shimoda M, Adachi T, Shimizu H, Weiss SJ, et al (2012) Neutrophil-derived matrix metalloproteinase 9 triggers acute aortic dissection. Circulation 126: 3070–3080." 10.1161/CIRCULATIONAHA.112.097097 23136157

[38] Remus EW, O'Donnell RE Jr., Rafferty K, Weiss D, Joseph G, Csiszar K, et al (2012) The role of lysyl oxidase family members in the stabilization of abdominal aortic aneurysms. Am J Physiol Heart Circ Physiol 303: H1067–H1075. 10.1152/ajpheart.00217.2012 22904155 PMC3469640

[39] Rodriguez C, Martinez-Gonzalez J, Raposo B, Alcudia JF, Guadall A, Badimon L (2008) Regulation of lysyl oxidase in vascular cells: lysyl oxidase as a new player in cardiovascular diseases. Cardiovasc Res 79: 7–13. 10.1093/cvr/cvn102 18469024

