# Supplementary figures and images for: Effect of Lysyl Oxidase Inhibition on Angiotensin II-Induced Arterial Hypertension, Remodeling, and Stiffness
Source: PLoS One. 2015 Apr 13;10(4):e0124013. doi: 10.1371/journal.pone.0124013 (PMC4395147; doi:10.1371/journal.pone.0124013)

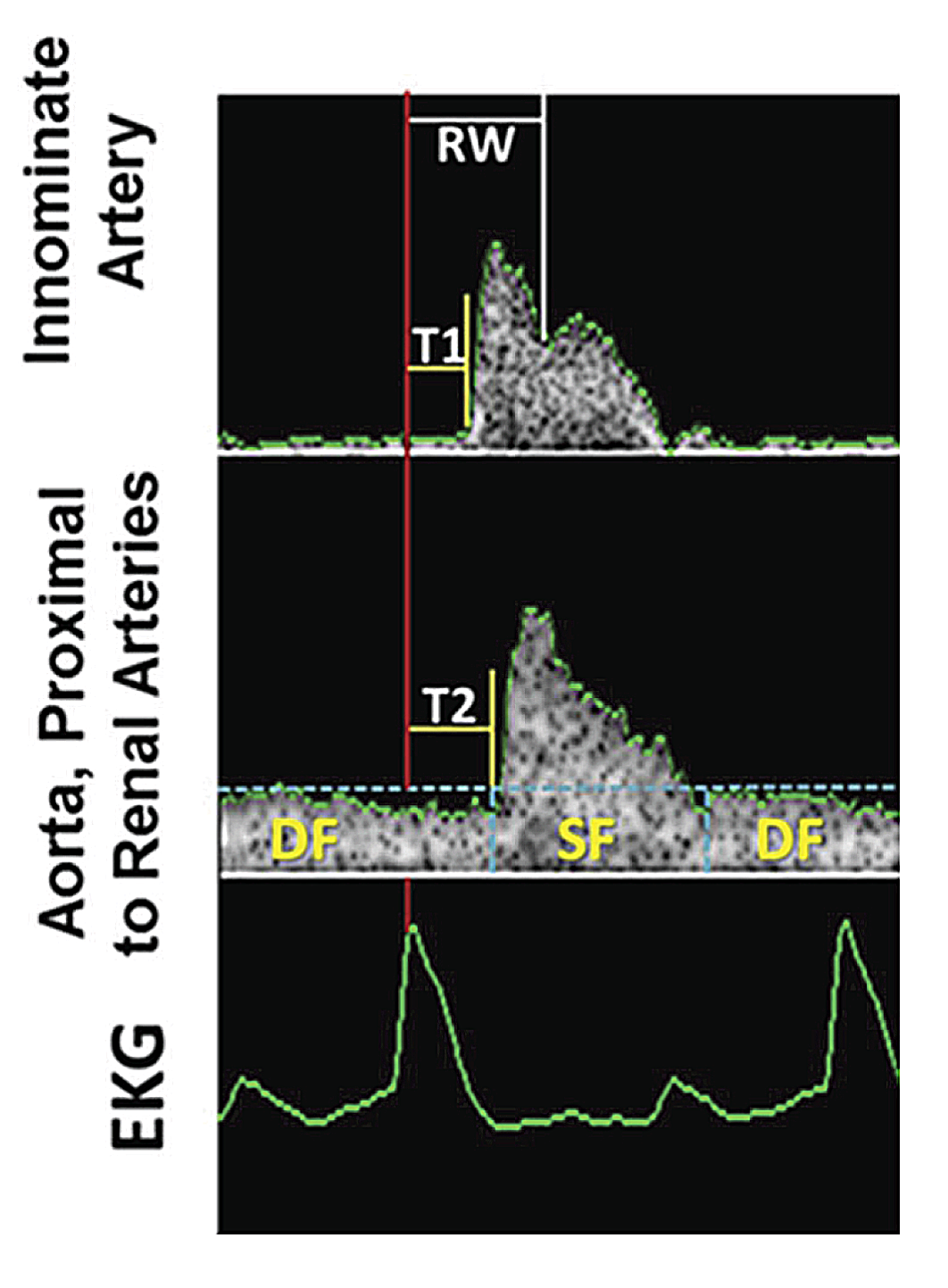

Supplement: S1 Fig — Transit times T1 and T2 were determined by the time from the peak of the EKG-R wave and the first pixel increase on their respective flow tracing. The distance between the innominate artery and the abdominal aorta just superior to the renal bifurcation was and T2 minus T1 was used to determine PWV. RW transit time was measured from the peak of the EKG-R wave and the nadir of the innominate artery flow Doppler. The DFF was computed by the area of diastole flow (DF) compared with the area of systolic flow (SF). (TIF) [file pone.0124013.s001.tif]

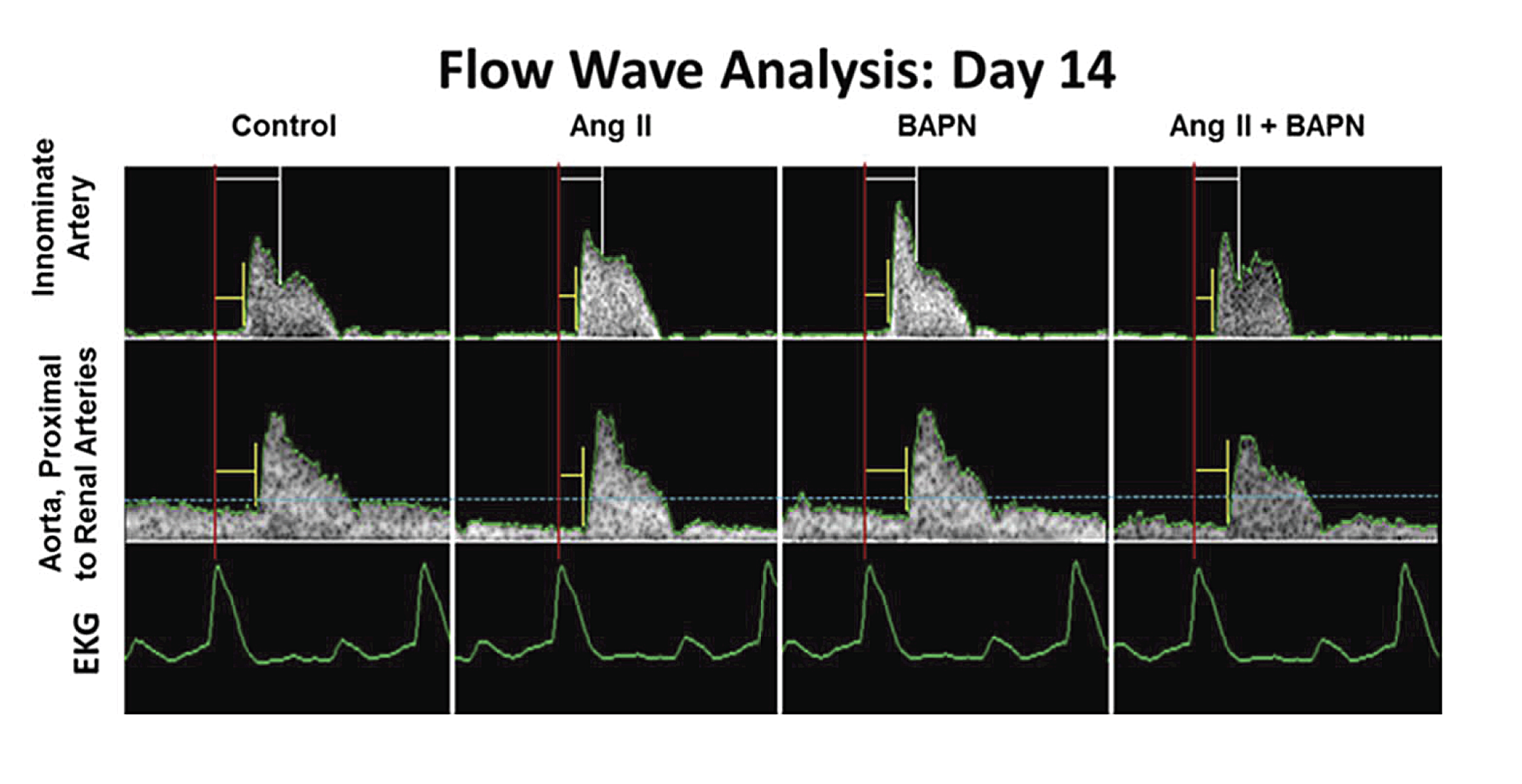

Supplement: S2 Fig — The red line represents the peak EKG-R wave. The yellow lines are the transit times from the R-wave and the first pixel increase in the flow tracing. The white line represents the nadir of the flow tracing in the innominate artery for the measurement of the RW transit time. The dotted blue line is the diastolic flow in the control aorta proximal to the renal arteries. (TIF) [file pone.0124013.s002.tif]
