# Supplementary material for: Effect of Lysyl Oxidase Inhibition on Angiotensin II-Induced Arterial Hypertension, Remodeling, and Stiffness
Source: PLoS One. 2015 Apr 13;10(4):e0124013. doi: 10.1371/journal.pone.0124013 (PMC4395147; doi:10.1371/journal.pone.0124013)
Supplement: S1 Methods — (DOCX) [file pone.0124013.s003.docx]

**METHODS Supplement for:**

**Effect of Lysyl Oxidase Inhibition on Angiotensin II-Induced Arterial Hypertension, Remodeling, and Stiffness**

Lance S. Eberson^1,3^, Pablo Sanchez^1,3^, Beenish A. Majeed^2,3^, Supannikar Tawinwung^2,3^, Timothy W. Secomb^1^, Douglas F. Larson^1,2,3^*

**^1^** Department of Physiology, The University of Arizona, Tucson, AZ, USA

**^2^** Department of Pharmacology, The University of Arizona, Tucson, AZ, USA

**^3^** Sarver Heart Center, College of Medicine, The University of Arizona, Tucson, AZ, USA

**Methods**

**Measurement of vascular stiffness in vivo.**

**Pulse Wave Velocity**

As illustrated in S1 Fig. the transit time difference (T2-T1) between the innominate artery and the distal aorta superior to the renal bifurcation was determined with respect to the EKG R-wave. The linear distance between the innominate and renal arteries was measured under direct view with *ex vivo* dissection and the distance divided by the transit time difference to compute the PWV.

**Estimation of Wall Mechanical Properties using the PWV**

The Moens-Korteweg equation for pulse wave velocity, c, is stated as:

c=(Eh/2Rρ)^1/2^ (Formula 1)

where E is the Young’s modulus, h is the wall thickness, ρ is the density of blood, and R is the vessel radius [1]. In order to calculate wall stiffness, Eh, from a given pulse wave velocity, the Moens-Korteweg equation was rearranged:

Eh=2Rρc^2^ (Formula 2)

The Young’s modulus, E, was then deduced using the data on wall thickness, h (including aortic intima, media, and adventitial thickness). Because the arterial wall structure is highly heterogeneous, the values of Eh and E obtained by this method represent effective values for overall wall properties.

To compute the diastolic flow fraction (DFF), S1 Fig. shows that the systolic and diastolic velocity-time-integral (VTI) were acquired to determine systolic flow (SF) and diastolic flow (DF) separately superior to the renal bifurcation. The systolic VTI (SVTI) and aortic diameter and diastolic VTI (DVTI) were computed individually. M-mode was used to define the systolic and diastolic diameter (SD) and (DD) respectively. These values were then used to determine the total diastolic flow fraction (DFF).

SF= (SD2*0.785*SVTI*HR)/1000 (Formula 3)

DF=(DD2*0.785*DVTI*HR)/1000 (Formula 4)

DFF=(DF/DF+SF)*100 (Formula 5)

To determine the central aortic reflection wave (RW) transit time of the forward flow nadir as illustrated in S1 Fig. was determined with respect to the EKG R-wave as described by Weber et al [2]. S2 Fig. shows the relative differences of arterial waveforms among the treatment groups.

**References**

1. Nichols, W. W., O"Rourke, M. F, Vlachopoulos, C., Hoeks, A. P., and Reneman, R. S. (2011) McDonald's Blood Flow in Arteries Theoretical, Experimental and clinical Principles. London: Hodder Arnold. 66 p.

2. Weber T, Wassertheurer S, Rammer M, Haiden A, Hametner B, Eber B (2012) Wave reflections, assessed with a novel method for pulse wave separation, are associated with end-organ damage and clinical outcomes. Hypertension 60: 534-541. HYPERTENSIONAHA.112.194571 [pii];10.1161/HYPERTENSIONAHA.112.194571 [doi].
